# Wrist disarticulation associated with Monteggia fracture

**DOI:** 10.11604/pamj.2015.21.320.6679

**Published:** 2015-08-31

**Authors:** Monsef El Abdi, Jonathan Bassinga

**Affiliations:** 1 Department of Orthopaedic Surgery «1», Military Hospital of Instruction Mohammed V, Rabat, Morocco

**Keywords:** Monteggia fracture, wrist disarticulation, ipsilateral elbow

## Image in medicine

A 50-year-old man, right-handed farmer, was admitted to the emergency department after undergoing an agriculture accident. Physical examination on admission revealed a left wrist disarticulation with a deformity of the ipsilateral elbow and forearm (A and B). Plain radiography showed a fracture of the ulna shaft as well as dislocation of the radial head. This radiological finding, also called Monteggia fracture-dislocation, was associated with a radiocarpal amputation of wrist-joint (C and D). Monteggia fracture-dislocation with wrist amputation is an uncommon condition. The patient was treated successfully with closed reduction of the elbow dislocation and internal fixation of the ulna fracture. The evolution was satisfactory.

**Figure 1:**
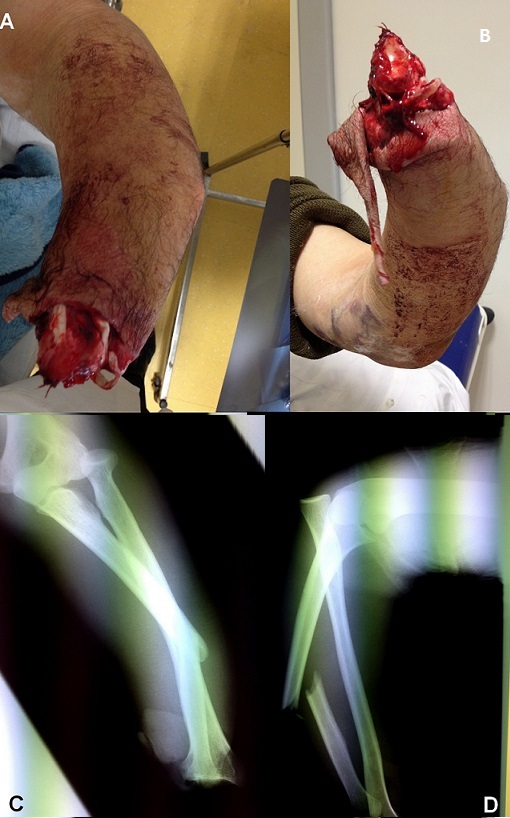
(A and B) clinical features in patient at admission showing wrist disarticulation with a deformition of the elbow and forearm; (C and D) radiography objectified a Monteggia fracture-dislocation with a radiocarpal amputation of wrist-joint

